# Influence at work is a key factor for mental health – but what do contemporary employees in knowledge and relational work mean by “influence at work”?

**DOI:** 10.1080/17482631.2022.2054513

**Published:** 2022-03-30

**Authors:** Malene Friis Andersen, Peter Aske Svendsen, Karina Nielsen, Svend Brinkmann, Reiner Rugulies, Ida Elisabeth Huitfeldt Madsen

**Affiliations:** a Department of Psychosocial Work Environment, National Research Centre for the Working Environment, Copenhagen, Denmark; b Department of Management School, Sheffield University, Sheffield, UK; c Department of Communication and Psychology, University of Aalborg, Denmark

**Keywords:** Influence, work, job-strain, well-being, organization, stress

## Abstract

**Purpose:**

Common mental health problems are a substantial burden in many western countries. Studies have pointed out that work related factors can both increase and decrease the risk of developing mental health problems. Influence at work is a key factor relating the psychosocial work environment to employees mental health. However, little is known regarding how contemporary employees experience and understand influence at work. The purpose of this study is to explore this in depth.

**Methods:**

We conducted semi-structured interviews with 59 employees in knowledge and relational work and analysed the data using principles from Interpretative Phenomenological Analysis (IPA).

**Findings:**

TWe identified three themes each consisting of two interrelated parts, where the second part describes the consequences of the identified type of influence for employees: 1) work tasks and performance, 2) relations and belonging, 3) identity and becoming.

**Conclusions:**

The interviewed employees had a multifaceted understanding of influence at work and that influence at work mattered to them in different but important ways. Our hope is that managers, employees and consultants will be inspired by the three themes when designing work tasks, organizations and interventions in order to increase the level of influence and thereby help enhance the mental well-being of employees.

## Introduction

Common mental health problems such as stress, depression and anxiety are a substantial burden in many western countries (OECD, 2018) The development of mental health problems is complex and the causes are multifactorial involving both biological, psychological and social factors (Engel, 1977). However, studies have pointed to working life as a contributing factor for adults who are part of the workforce. One British study estimated that work-related stress, depression and anxiety in 2016–2017 accounted for 40% of work-related illnesses and 49% of all lost work days (Health and Safety Executive, 2017). In a Danish study of 34.800 Danish randomly selected employees, 15% report that they felt stressed. Among these, 53% reported that work was the cause of their stress, while 42% reported both work and private life as the causes (NFA, 2017). To prevent mental health problems it is vital to gain a deeper understanding of what aspects of working life affect the mental health of employees.

Studies have found that one of the major negative factors in working life is perceived lack of influence at work leading to feelings of powerlessness, helplessness (Czuba et al., 2019), and compassion fatigue (Norrman Harling et al., 2020). Based on a multitude of quantitative studies, reviews have concluded that influence at work is one of the most important factors in the psychosocial working conditions to employees’ mental health (Kivimäki et al., 2019; Madsen et al., 2017). Karasek and Theorell’s job strain model from 1979 has a prominent role in occupational health research in relation to when it comes to health effects of low influence at work (Fransson et al., 2012; Karasek, 1979; Karasek & Theorell, 1990). The influential model focuses on two job characteristics: Job demands (e.g., workload, work pace) and job control (influence on task solving and development opportunities in work). Recent studies have shown that especially the control or influence dimension of the job strain model is associated with mental health (Madsen et al., 2017; Theorell et al., 2016, 2015). Low job control is also one of only three working conditions graded in a previous review as having moderate evidence for being related to clinical depression (Theorell et al., 2015). As the association between influence at work and mental health is well established it is vital to understand the mechanisms linking the two and understanding how to increase employee influence on their working life. This could help reduce the risk of work-related health problems and low well-being. However, even though knowledge on how contemporary employees perceive influence at work and what meaning they ascribe to influence at work is essential, it is missing from the extant literature. One reason for this knowledge gap is that influence in occupational health research is mostly studied quantitatively by means of especially two questionnaires: JCQ (Job Content Questionnaire) and DCQ (Demand Control Questionnaire; De Jonge & Kompier, 1997; Karasek, 1979; Väänänen & Toivanen, 2018). The domination of quantitative measures and the wide use of the job-strain model have led to critics arguing that the job-strain model is outdated, and repeating previous methodological approaches and psychometric scales can even: *“ … inhibit innovation, both conceptually and methodologically”*(Väänänen & Toivanen, 2018). In an Editorial Väänänen and Toivanen (2018) call for an update of conceptual and methodological approaches to influence through qualitative empirical research in order to update our knowledge on influence in contemporary work (Väänänen & Toivanen, 2018). They argue that the labour market has changed substantially and there has been a shift in the type and organization of jobs since the development of the Job-strain model, particularly in post-industrial and high-income countries (Väänänen & Toivanen, 2018). More employees now work in white collar jobs with knowledge, symbols, services and care rather than in blue collar jobs in industry or agriculture as was the case in the 60s and 70s (De Jonge & Kompier, 1997; Väänänen & Toivanen, 2018). Also, organizations tend to have flatter hierarchies and the amount of team-work and flexible work arrangements has increased (Edmondson & Lei, 2014; Väänänen & Toivanen, 2018). These changes in jobs and organizations may have led to changes in how employees experience influence at work and what aspects of their working life they perceive as important to have influence on. The present study follows Väänänen and Toivanens call for an update of conceptual and methodological approaches to influence in contemporary work through qualitative empirical research on employee’s experiences of influence (Väänänen & Toivanen, 2018). We apply an inductive analytical approach based on comprehensive data from semi-structured interviews with 59 employees working with tasks related to knowledge, care and relations to answer the following research questions:

- What do contemporary employees in knowledge and relational work associate with influence at work?
- What importance does influence at work have for these contemporary employees?

We believe that the answers to these questions can also help managers, consultants and organizations to organize tasks and work processes in ways that increase employeesinfluence at work and thereby potentially reduce their risk of mental health problems.

## Methods

This study presents results from a comprehensive qualitative research project on influence at contemporary work. The first author conducted 59 qualitative interviews in two different organizations, one organization was a large pharmaceutical company, and the other was a department in public psychiatric care. The interviews were conducted between March 2018 and December 2018. To secure the anonymity of the organizations and the interviewees we call the organizations Pharma Solution and Therapy Garden. The two organizations were chosen as information-oriented cases (Flyvbjerg, 2006) representing knowledge-intensive work and relational-intensive work respectively. In both organizations work was organized in teams and there was a high degree of cooperation between employees as most of the work tasks demanded contributions and inputs from specialists with different educational backgrounds. The first author had initial meetings with managers and employees from the two organizations before the final selection of the two to secure that they represented suitable cases exemplifying knowledge- and relation-intensive work and organizations. Pharma Solution is a big international pharma company with over 15,000 employees. The interviewees were recruited from three teams consisting of 12–19 employees. The four teams all worked in the Pharma Solution headquarter and their main tasks were planning and evaluating clinical trials world wide. They all had Masters level university degrees—typically in mathematics or health and medical sciences—and worked with conducting, analysing and reporting clinical studies.

Therapy Garden is part of the public psychiatric health care system with more than 4,000 employees. The interviewees were recruited from four teams consisting of 9–16 employees. The four teams worked at the same site in a psychiatric clinic. Employees in these teams were healthcare professionals with Bachelor level degrees (e.g., nurse, social worker) and supplementary psychotherapeutic educations or Master level degrees (e.g., psychologists, psychiatrists). Their core tasks were specialized examination and treatment of individuals with psychiatric disorders.

### Data collection

Employees in the participating teams received an email from the first author with an invitation to participate in an individual interview. It was emphasized that participation was voluntary and that the participants could withdraw from the study at any time. Anonymity was guaranteed. Twenty-three employees from the three teams in Pharma Solution accepted the invitation and thirty-six employees from the four teams in Therapy Garden accepted the invitation. In this way, about 50% from the teams in both organizations chose to participate in the interview. The interviewees were between 26–65 years old and approximately 60% were females. This distribution reflects the team distribution of age and gender. Interviews were conducted by the first author and lasted approximately one hour. The starting point for the interviews was a semi-structured interview guide that comprised overall themes such as: What do you associate with influence in work? What is important for you to have influence on in your work? What does it mean to you (in terms of job satisfaction, meaning of work) to have influence at work? How does it affect you to have/not to have influence? No definition or conceptualization of influence was provided to the participants. Instead, they were asked throughout the interview to explain their experience with and perception of influence at work.

In Denmark, an approval from Ethical Committee is not required as this study did not include biomedical research, but the study was registered with the Danish Data Protection Agency and approved by this (no. 2015–57-0074). All authors followed the principles of the Declaration of Helsinki.

### Data processing and analysis

In total, 57 of the 59 interviews were audiotaped and transcribed verbatim. Two interviewees felt uncomfortable with audio recording and their interviews were analysed based on interview notes written down during interviews.

Theoretically and methodically this study is inspired by Interpretative Phenomenological Analysis (IPA; Smith, 2004; Smith et al., 2009). IPA is based on phenomenology (an approach to the study of human experience from a first-person perspective), hermeneutics (the theory of interpretation) and symbolic-interactionism (focusing on the meanings people attach to situations which can only be accessed through interpretation; Smith et al., 2009). In IPA it is assumed that it is possible to extract generic and general theories on the basis of analysis of qualitative interviews. IPA is inductive and concerned with exploring people’s lived experiences and how they make sense of important themes and subjects in life (Smith, 2004; Smith et al., 2009). Due to the large amount of data, a slightly adapted version of IPA was applied: The 59 interviews were all read and re-read by the first and second author and units of meaning were identified as central aspects of the participants’ experiences. Based on ten randomly selected interviews (five from each organization), the first author developed hierarchic level 1 thematic codes, which more descriptively deal with the subjects the interviewees reflect on (what Tracy (2013) terms “the central themes present in the data” (Tracy, 2013)). Subsequently all the interviews were coded in the data system NVivo 11 on the basis of the codes developed and data were read and analysed across the selected codes by the first author. Using IPA, we identified the three overarching themes and these have continuously been discussed in the group of authors. In the following result section they will be presented and unfolded. As there was a considerable overlap between the employees’ experiences from the two organizations, we present them together and integrated in the result section.

## Results

The analysis identified three overarching themes. The three themes consist of two interrelated parts: The first part illuminates what in their working life the interviewees experienced as important in relation to influence at work. The second part describes the consequences of the identified type of influence for the interviewees. The three themes are as follows: 1) Influence and work tasks—Performing, 2) Influence and relations—Belonging, 3) Influence and identity—Becoming. Table I “Results—Type and Consequences of Influence” sums up the results of each theme and the interrelatedness of the two parts.

**Table I.** Results—Type and consequences of influence

Type of influence	Consequences of the type of influence
*Work tasks* The employees experience influence when they have a say in • how to solve tasks • how to prioritize the tasks • in which order the tasks are solved • when the tasks are solved	*Performing* Enables the employees to perform by • securing a better fit between method and task • focusing on the most important and urgent tasks • balancing work demands and cognitive resources and energy • improving work-life balance
*Relations* The employees experience influence when they • exchange views and knowledge • have a voice • feel involved in the daily work processes • affect others and are affected by others	*Belonging* Instils a feeling of • being a legitimate part of what is going on • having a significant position in a meaningful whole • feeling worthy and important to others
*Identity* The employees experience influence as • an personal value • an existential condition • related to self-worth and self-esteem	*Becoming* The amount of influence determines who the employees become: • low influence: becoming non-human, an object like a pawn, a robot or an animal • High influence: becoming a subject, initiating a feeling of being alive, active and a unique human being

## The three themes are presented in the following section

### Influence and work tasks—Performing

The interviewed employees found it important to have influence on their core work tasks. They were concerned with having influence on how they solved their tasks, in which order they solved their tasks, how they prioritized their tasks, and when they solved the tasks.

One employee puts it this way:

To me influence means influence on how I plan my work, how I prioritize the tasks. I mean a kind of freedom also concerning solving the tasks. And confidence that as an employee I do what it takes in a given situation sometimes without fully following formal structures or guidance or instructions. That a degree of freedom is allowed when it comes to the choice of solution of a particular task. (Nancy, Therapy Garden)

As shown in the quote, the employee finds it important to have a degree of influence on how to prioritize the tasks and how a particular work task is solved. Having influence especially on how tasks were solved, was generally appreciated among interviewed employees in both organizations and was primarily related to the methods used to solve the tasks. Working in complex organizations with high interdependence between employees and departments the interviewees acknowledged the need for corporate standardization of work processes and work tasks. Despite accepting this, the majority of the employees stressed the importance of having sufficient influence to make their own decisions about which methods to use and when was considered relevant:

In our statistics team we try to align our methods. But we are also free to do things our own way if we think this makes sense and is best for our projects. Of course it is pleasant and motivating that it is like that. I would find it demotivating if everything had to be placed in a sort of standardized tool box because, yes, exactly because it is statistics, it is not an exact science (Michaela, Pharma Solution)

Given the different core tasks in Pharma Solution and Therapy Garden, the employees in the two organizations differed on which specific methods they considered important. However, the essence of this need is similar, as can be seen in the following quote by a therapists from Therapy Gardens:

To have a say and be able to decide: well, this [therapeutical method] makes sense to use in the treatment. We work cognitively but we don’t only work cognitively. At the moment, I take a schema therapy education because we would like to introduce more schema therapy into the treatment of those who suffer from slightly more severe personality disorders. And it is great to have a say in your work so you work with methods you think are meaningful (Amira, Therapy Garden)

Evidently, it was important for employees in both organizations to have sufficient influence to be able to adapt their work methods to the specific work task. This had much to do with the interviewees being concerned with delivering good quality work. It was important for them to solve their work tasks in a satisfying manner, and to be able to choose which methods to use to solve the work tasks was a significant part of this.

When asked what was important to have influence on, the majority of employees also mentioned having a say in *when* they solved which tasks. This flexibility helped them to alternate during the day and week between demanding and complex tasks and routine tasks. This was important in order to create a good balance between work demands and their cognitive resources and energy, which they experienced could vary during the workday and over a 5-day working week. A number of interviewees also mentioned having partly flexible work hours:

It [flexible work hours] makes you feel that you are in a way your own master. That is great. And it is also nice that the system doesn’t insist on deciding just because the system wants to decide. So they [the management] say, “well, as long as you do your own things. That is really really nice, I think.” (Kim, Therapy Garden)

Influence on their working schedule helped employees achieve a better work-life balance as they could balance e.g., family responsibilities.

### Influence and relations—Belonging

Both organizations were characterized by high interdependence between employees, and the contributions of employees with different professional backgrounds was often necessary to solve the complex tasks. The interviewed employees worked in teams where they depended on exchanging input, decisions and knowledge to succeed with their work tasks and contribute to the work process. One employee elaborates on how they cooperate:

Everybody is allowed to have a say. Of course we may disagree, but then we discuss it and present our views and then we decide which solution is best. To me it is really, really important that everybody understands that it is a common goal: If we get there it is a common victory for us. It is not me who achieve this it is us who achieve it. (Steve, Pharma Solution)

It was through discussions, the exchange of views and knowledge that employees felt they had influence. When asked how they experienced influence at work, interviewed employees in both organizations spontaneously mentioned being seen, heard, having a voice, and being part of the daily work processes:

I think influence is a feeling of being seen and heard. It is give and take. It is not so important if I decide as long as I am heard. Influence can be in the structures we have during the week, for example, team meetings and supervision, the daily routines, so to speak. To feel that you are part of what is going on is influence to me (Brandon, Therapy Garden)

This interrelatedness in contemporary knowledge and relational work may explain why most employees frequently referred to influence as something that was shaped and formed in relation to co-workers and managers. When the interviewed employees experienced they could contribute positively through their views, knowledge and know-how it increased their feeling of having a significant and important position: they belonged—to the team and the organization. In the following two quotations, interviewees give specific examples of experiencing influence by both giving and receiving views, knowledge and experience. As can be seen this contributes to the employees´ feeling of influence in relations and thus the feeling of belonging. The first quote points to the importance of giving, and the second points to the importance of receiving knowledge:

I was at a meeting the other day where we discussed how to write clinical outcomes. When we returned to and used what I had suggested earlier in the meeting I felt like this “I have a right to be here, yes, I belong (James, Pharma Solution)

If there is someone who spends time on informing me of something it also means that I am important enough to receive this information and for them to spend their time on giving it to me. It makes me feel that I am not just some pawn somewhere and, yes, that I am worthy of it (Brenda, Pharma Solution)

Having influence on the work process by giving and receiving knowledge and information generates interviewees’ feeling of belonging and being worthy of belonging. A feeling that was very important for the employees. Influence for them was not to make headstrong decisions but instead to be affected by others and affect others in return through mutual exchange in dynamic processes:

You are comfortable if you feel you are part of something and you have a voice in that context. And that voice is – well, now we return to the music metaphor, because to me the optimal influence scenario would be where the orchestra plays and all the instruments are important and one doesn’t work without the other. I mean, the concert won’t be a success if the triangle isn’t there. To me, influence is being part of a meaningful whole but not necessarily playing some significant part ….I am happy to leave the solo violin to others. But I expect the solo violinist to consider herself part of the whole orchestra (Madison, Therapy Garden)

According to this section, the interviewees also understood influence as a relational phenomenon associated with mutual exchange with co-workers and managers. They experienced influence when they gave and received information and knowledge, had a voice and experienced that others—including the solo violinist—listened and took their voices seriously. When this happened, the interviewees felt they belonged and that they were an important and worthy part of the team and organization.

### Influence and identity—Becoming

Several employees stressed that influence at work not only affected their ability to solve their tasks successfully in cooperation with colleagues and managers. Influence at work was an essential value in itself and significant for their job satisfaction:

My freedom is extremely important to me. Or my influence. I mean to be able to do my job as I please. It is very important to me … … It is also part of my job satisfaction to be able to do it that way. It is extremely valuable to me (Jacob, Pharma Solution)

Employees also related influence in work to their self-perception and self-understanding:

It [influence] is a way of feeling that you exist, that you are important, that you, well, are alive … That you are not some pawn to be moved around (Alice, Therapy Garden)

This quotation shows how an employee relates influence to a feeling of existing and being alive. Influence seems to play a central role in whether the employees experience they are—and are treated as—active subjects that act themselves rather than as passive objects that others act upon. Across both organizations we found a tendency for employees to equate not having influence at work with being non-human: a pawn, a robot, a machine or an animal..

When you work here you shouldn’t be too much of a robot. And robot means that you are on some sort of automatic pilot: you don’t invest too much, you don’t think too much, you are not too critical, you don’t take a stand, you don’t have too much influence. It strikes me that a robot has no influence. A robot has absolutely no influence! (Emma, Therapy Garden)

The employee continues:

Influence, I think it is about my self-esteem or self-worth. It means something to my self-perception and my way to be in the world. I mean, if I had a dog I would think: “It shouldn’t have any bloody influence”. But as soon as it is about human beings, I think you need to have influence to be a human being. Because you are not human if you don’t have influence. And it creates this feeling that you might as well be a machine. I think, that’s the way it is for me. (Emma, Therapy Garden)

As can be seen from the quotations the experience of influence affects how the employees think and feel about themselves. Employees mentioned influence as a necessary condition for active and critical thinking and engagement in their work process. Employees did not only relate influence to their work and their professional identity—but also in relation to their general self-understanding, self-worth and way of being in the world. Influence is needed in order to be and feel like a human, the employee states in the above quote. According to the interviewees, influence can thus be understood as an existential condition that differentiates humans from non-humans. Interviewees experienced that their feeling of being acknowledged and treated as unique human beings could be disrupted if their influence was low or they did not feel they were involved in organizational change processes:

If management think we are building blocks, and all have the same color or can quickly be painted a different color it creates frustrations. Because we are not all alike or have the same color or can be painted overnight (Luke, Pharma Solution)

## Discussion

The present study examined influence from the perspective of contemporary employees working with knowledge and relations. We have explored what parts of their working life they experienced as important to have influence on and ways influence matters to them. We found that the interviewed employees had a multifaceted understanding of influence at work and that influence at work mattered to them in different but very important ways. Our analysis identified three themes each consisting of two interrelated parts: 1) Influence and work tasks—Performing, 2) Influence and relations—Belonging, 3) Influence and identity—Becoming. In this section, we will discuss our results in relation to existing literature and in this way also explore which mechanisms might explain the relationship between influence and mental well-being.

The interviewees emphasized the importance of having influence on which methods they used, how they prioritized their tasks and their working time. Our findings are in line with previous studies showing that the possibility to choose how to solve tasks and decide in which order to do them is an important dimension of influence in work (Faturochman., 1997; Wilkinson et al., 2010). Several studies show how the feeling of having sufficient influence to engage with work tasks in a meaningful manner benefits employee job satisfaction and experience of meaning (Sasser & Sørensen, 2016; Semmer et al., 2019). Our study suggests that the experience of influence on work tasks increases the employees’ experience of being able to perform and the ability to deliver high quality work. Previous studies have shown employees can become frustrated, stressed and develop compassion fatigue if they feel barred from solving their work tasks competently (Johansson & Theorell, 2003; Norrman Harling et al., 2020; Sasser & Sørensen, 2016). Our results unfold some of the reasons why a high degree of influence seems to be necessary for contemporary employees like knowledge and relational workers. There is general consensus that globalization and the technological revolution have increased the complexity of work tasks and therefore also the employees’ need to be flexible and agile (Alvesson, 2004). New technologies, changes in the market, new knowledge and frequent organizational changes make it difficult to develop standardized solutions and methods that fit all tasks and that can be used at all times. This might be one of the explanations why the interviewees in our study experience that influence plays a central role in their ability to perform and solve their work tasks well—they simply need a high degree of influence to be flexible and develop the best solutions to solve the changing work tasks and to navigate in the rapidly changing work context.

In the result section we have also shown that the employees found it important to have some influence on when the task was solved as this made it easier for them to balance work demands and their resources and energy and to improve their work-life balance. The positive effect of having influence on work hours is also documented in other studies showing that flexible work conditions have a positive effect on mental health (K Joyce et al., 2010). However, flexibility comes with a potential price. Studies have shown that flexibility can lead to increased work intensification and work hours and it has been suggested that this can be explained by employees trading flexibility for effort (Kelliher & Anderson 2010). We need to be aware of both positive and negative effects of this type of influence.

We have shown that the interviewees also experienced influence as something that was created and shaped in relation to and with co-workers and managers. The interviewees felt they had influence when they gave and received knowledge and information, were able to affect the work process and had a say. This evokes the concept “voice behavior”, defined as employees’ sharing ideas, information, and thoughts on improvement of work tasks and the organization (Dyne et al., 2003). It has been suggested that the experience of having influence is a possible precondition for voice behaviour: One needs to believe that it is possible to affect colleagues or managers to practice voice behaviour (LePine & Van Dyne, 1998; Tornau & Frese, 2013). Our results support the notion that the experience of having influence to affect colleagues and work processes can increase employees’ voice behaviour. But our results also suggest the reverse relation: that practicing voice behaviour contributes to the experience of having influence. The experience of influence at work is therefore also created in and through voice behaviour and the quality and character of work relations might decide if it is possible to practice voice behaviour. The relationship between relations, influence and voice behaviour might be a more reciprocal and dynamic relation than suggested in the quantitative studies (LePine & Van Dyne, 1998; Tornau & Frese, 2013).

A promising concept to study employee influence as reciprocal and dynamic relations is “tied autonomy” (Väänänen & Toivanen, 2018). This concept suggests that contemporary employees working with knowledge and relations have a high degree of influence but at the same time they are utterly dependent on co-workers in the work process and task solution who also have a high degree of influence. As a consequence the individual employee influence is embedded in “multiple social and organizational relationships” (Väänänen & Toivanen, 2018). Another recent study argues that influence is created, increased or decreased through collaboration with other employees in the organization, and suggests that the measurement of influence at work primarily at the individual level limits our understanding of the phenomenon (Wåhlin-Jacobsen, 2018).

Our empirical study supports this theorizing of influence as a relational, reciprocal, and dynamic. Additionally, we have illuminated that when the interviewees participated in influence related exchange activities with colleagues and managers they felt they belonged. The identified link between influence, exchanging and belonging can be understood with the help of social philosopher, Axel Honneth’s (2003) theory of recognition. Honneth’s concept of intersubjective dependence, defined as the importance of being recognized as a person who can contribute to and affect the group through his/her qualities and who is important enough for the group to relate to and affect (Honneth & Willig, 2003). If we examine influence in this framing it becomes a precondition for belonging and feeling worthy of belonging—feelings that are important for our mental health. Epidemiological studies have shown that the opposite feelings such as loneliness, isolation and not being part of a community are major risk factors for mental health problems such as depression, stress and sleep problems (Mushtaq et al., 2014; Park et al., 2004). Our study has shown how influence and belonging are related and we have hereby identified one—out of many—explanations for the relationship between influence at work and mental health—an explanation that to our knowledge is not part of the existing discussion of influence in occupational health research to day.

We found that interviewees’ experiences of influence affected their self-understanding as active subjects or passive objects. Thus there seems to be a significant spill-over effect between the interviewees’ experience of influence at work and their identity (Erikson & Erikson, 1998). This is an interesting finding in relation to the literature on work identity (Knez, 2016; Walsh & Gordon, 2008) that is based on the assumption that work identity can be described as generally separated from personal identity (Knez, 2016; O’Connor et al., 2008). Our study challenges that assumption as we have found that the interviewees do not maintain this separation. However, the degree of influence at their disposal at work affects their perception of themselves. In a theoretical paper Al Gini (1998) also challenges the idea of work identity as a limited and isolated phenomenon. Gini argues that work is central to “what we’ll become”, and he therefore stresses that we should be very careful about what we do for a living and where we work (Gini, 1998): “The lessons we learn in our work and at work become the metaphors we apply to life and others, and the means by which we digest the world” (Gini, 1998). We found that influence at work is important in relation to the metaphors that the interviewees use to describe and understand themselves—not only as employees but also as humans. And we argue that this spill-over effect has intensified for contemporary employees working with knowledge and relations. Researchers within the theoretical school “cognitive capitalism” (Alvesson & Willmott, 2002; Hirschhorn, 1998) have argued that a considerable part of the identity of contemporary employees is embedded in their work (Alvesson & Willmott, 2002; Hirschhorn, 1998). It is not a choice for employees working with knowledge, relations and care to not use competences and qualities drawn from their personality to solve work tasks. Employees use social and analytical skills, creativity, and cooperative abilities to execute tasks. A consequence of this is increasing inseparability of work and identity as the identity of the employees becomes a vital production factor (Hirschhorn, 1998; Pedersen, 2009). The spillover effect from professional to personal identity identified in our study should also be understood in this context. The strong link between work and identity for contemporary employees may intensify the importance of influence at work for their identity and for who they become. However, this shown association between influence at work and becoming also calls for attention to inequalities at the labour market—maybe we are not equally free to “be very careful about where we work” as Gini recommends (1998). The level of influence at work is not equally distributed between different job groups where employees with no or low education have the lowest degree of influence (Clausen et al., 2019) and they also suffer more from job insecurity and unemployment than high educated employees (OECD, 2022). We need to give this more attention both in research, in practice and in labour market politics if we want to create equal opportunities for influence and thereby more equal opportunities for becoming active subjects in our own lives.

In the Discussion we have identified the importance of the three types of influence and have also briefly touched upon potential negative side-effects of a high degree of influence and we hope that future studies will look into potential conflicts and ambivalence in the employees regarding the identified types of influence. As mentioned in the Introduction influence is mostly studied quantitatively by means of the Job-strain model and the widely used questionnaires JCQ (Job Content Questionnaire, (Karasek et al., 1998)) and DCQ (Demand Control Questionnaire, (Karasek et al., 2007)) are shaped by this model. These questionnaires define and measure influence as being able to plan and decide how to solve tasks (Fransson et al., 2012) which is very much in line with our first theme ‘influence and work tasks—Performing´. However, the questionnaires have limited focus on the two additional themes found in this study. JCQ nevertheless includes one question regarding relations and influence (“I have much to say about what happens in my work”). Our hope is that future quantitative studies will be inspired by our thematization of influence and elaborate the questionnaires so they will be better suited to capture broader aspects of influence relevant for employees. We hope qualitative and quantitative researchers will be able to update the conceptual and methodological approaches to influence in cooperation.

## Limitations

Our study is not without limitations and the findings must be considered in this light. The interviewees have relatively long educations and work with knowledge and relations. Our findings might be specific to these types of jobs and cannot necessarily be generalized to employees working in other sections of the workforce. It would be highly relevant if other studies could test, validate or challenge our three themes. We therefore hope that future studies would look into these aspects focusing on employees working within the same and other types of jobs, e.g., industries and trades. Further, our study was conducted in a Scandinavian context. Studies have indicated that Scandinavian employees both experience and expect to have a higher degree of influence than employees in other European countries (Sørensen et al., 2015). We need research outside Scandinavia to determine to what extent our results are transferable to other cultures.

## Practical implications

We have pointed to three different parts of influence at work in modern working life that are important to consider when creating healthy work places that will contribute to increasing the mental health of contemporary employees. Our hope is that managers, employees and consultants will be inspired by the themes illuminated in this study when designing work processes, tasks and organizations so the level of influence can be increased in relevant and efficient ways. A recent review on workplace interventions for common mental disorders shows evidence that workplace interventions focusing on increasing employees’ influence on their working conditions enhance their mental well-being (S. Joyce et al., 2016). Be believe that the here presented deeper and broader understanding of influence can help guide organizational interventions and thereby hopefully enhance the positive effects of such interventions.

## Conclusion

Our study provides an in-depth exploration of contemporary employees’ experience of influence at work. In our article we have shown how influence at the workplace is important for contemporary employees regarding being able to perform and to fundamental psychological needs such as belonging and positive becoming as active subjects. We have illuminated the complexities of employees’ experiences of influence at work and we believe this knowledge can also help us understand the mechanisms more thoroughly of why influence at work is one of the work factors that affects the mental well-being of employees most.
